# A novel nomogram and risk classification system for predicting lymph node metastasis of breast mucinous carcinoma: A SEER‐based study

**DOI:** 10.1002/cam4.4804

**Published:** 2022-05-22

**Authors:** Shuang‐Ling Wu, Jun Da Gai, Xin Miao Yu, Xiaoyun Mao, Feng Jin

**Affiliations:** ^1^ Department of Surgical Oncology and Breast Surgery the First Affiliated Hospital of China Medical University Shenyang China; ^2^ Department of Pathology the First Affiliated Hospital of China Medical University Shenyang China

**Keywords:** lymph node metastasis, mucinous breast cancer, nomogram, surveillance, epidemiology and end results (SEER)

## Abstract

**Background:**

Mucinous breast cancer (MBC) is a rare disease, and patients with lymph node metastasis (LNM) have a poor prognosis. We aimed to explore the predictive factors of LNM and to construct a nomogram for predicting the risk of LNM and to identify the suitable axillary surgery for patients with diverse risks.

**Patients and Methods:**

Data were extracted from the Surveillance, Epidemiology, and End Results (SEER) database. Chi‐square and rank‐sum tests were used to analyze the differences between groups. Survival analysis was performed with Kaplan–Meier curves and log‐rank tests. Independent factor identification and nomogram construction were performed with logistic regression analysis. The nomogram was qualified with a discrimination and calibration plot. Propensity score matching was performed to balance the disparities between groups.

**Results:**

Patients with metastatic lymph nodes have a worse prognosis. Univariate and multivariate analyses indicated that tumor size, grade, and age were independent risk factors for LNM. The nomogram constructed with these three factors can predict the risk of LNM with high accuracy (AUC: 0.767, 95% CI: 0.697–0.838) and good calibration. Based on the nomogram, a risk classification system satisfactorily stratified the patients into 3 groups with diverse risks of LNM. In the low‐risk group, there were no significant differences between sentinel lymph node biopsy and no axillary surgery. In the middle‐ and high‐risk groups, both SLNB and axillary lymph node dissection were superior to no axillary surgery, with similar survival benefits.

**Conclusions:**

The nomogram based on tumor size, grade, and age could conveniently and accurately predict the risk of LNM in MBC and assist clinicians in optimizing surgical strategies.

## Graphical abstract

1. In this study, we observed that mucinous breast cancer with metastatic lymph nodes have a worse prognosis and identified tumor size, grade and age as independent risk factors of lymph node metastasis. 2. we constructed a nomogram with factors of tumor size, grade, age and developed a risk classification system for predicting the risk of lymph node metastasis. 3. Based on the nomogram and our classification, we identified optimal axillary surgery for patients in the low‐, middle‐ and high‐risk groups.

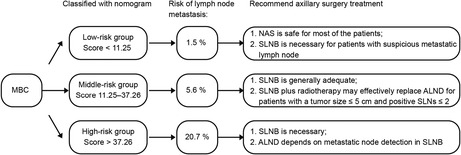

## 1 INTRODUCTION

Mucinous breast carcinoma (MBC) is a rare and special type of breast cancer that accounts for nearly 1–6% of all cases of primary breast cancers.^1^, ^2^, ^3^ Compared to most invasive breast cancers, MBC has special epidemiological, clinicopathological, and molecular features. MBC commonly occurs in elderly postmenopausal women,^4^ with a median age of 70 and is generally considered to have a lower grade, better differentiation, and lower rate of lymph node metastasis. In addition, MBC was observed to have higher expression of estrogen receptor (ER) and progesterone receptor (PR) and lower expression of human epidermal growth factor receptor‐2 (HER2).^5^, ^6^ In the majority of studies, patients with MBC usually have a better prognosis^7^, ^8^ and their 5‐year overall survival rate ranges from 95% to 98.9%,^9^, ^10^ and their 10‐year overall survival rate is as high as 90.4%.^1^ To date, some studies have evaluated the prognostic factors for MBC and proposed that lymph node metastasis, age, tumor size, tumor grade, and PR are all related to the patient survival rate. Among these factors, axillary lymph node status is deemed to be the most significant independent outcome predictor.^6^, ^10^, ^11^, ^12^, ^13^

Because of its low incidence and the limited number prospective studies, there is still a lack of clear recommendations about the clinical management of MBC, and treatments for MBC are mostly extrapolated from invasive breast cancer. In the current guidelines and clinical practice, surgery and adjuvant treatments are still the main therapeutic strategies for MBC. Regarding the surgical strategies, breast‐conserving surgery plus radiotherapy and mastectomy are both effective.^14^, ^15^ Regarding adjuvant therapy, no benefits of chemotherapy were observed in HR‐positive and node‐negative MBC, while for triple‐negative MBC, chemotherapy is still a choice in clinical practice, especially for advanced MBC.^16^, ^17^ Unlike chemotherapy, postoperative radiotherapy following breast‐conserving therapy was observed to be associated with a better prognosis of MBC among elderly patients.^18^ Additionally, endocrine therapy is generally recommended for HR‐positive MBC, especially for advanced cases,^19^, ^20^ although some research has suggested that the lack of PIK3CA mutations in mucinous carcinoma may make it different from luminal breast cancer.^21^ Aside from endocrine therapy, in recent years, some researchers have proposed that an anti‐HER2 treatment strategy might be valuable for HR‐positive, node‐positive, and HER2‐positive MBC.^22^, ^23^

Considering its favorable prognosis, some researchers have recommended identifying more optimal and less aggressive treatments for MBC. Recently, breast‐conserving therapy has been suggested for locoregional treatment of T1–2 stage MBC with a better prognosis instead of mastectomy.^14^ However, the treatment of axillary lymph nodes remains ambiguous. In light of the notable role of lymph node status in MBC prognosis, to better manage systematic treatments, assessing the lymph node status and applying appropriate axillary surgery are nonetheless crucial for patients diagnosed with MBC. In recent years, nomograms have been broadly used for the prediction of lymph node metastasis and patient prognosis.^24^, ^25^, ^26^ Therefore, in this study, we aimed to identify risk factors for lymph node metastasis of MBC with clinicopathological and demographic information from SEER. Then, a nomogram model was established to predict the risk of lymph node metastasis in MBC. Finally, we aimed to investigate how to decide upon the most suitable axillary surgery for MBC with different metastatic risks, which will be useful in the clinical management of axillary lymph node resection.

## 2 MATERIALS AND METHODS

### 2.1 Database and patient selection

17,996 patients enrolled in this study were acquired from the Surveillance, Epidemiology, and End Results (SEER) database (http://seer.cancer.gov/), which is sponsored by the National Cancer Institution. The SEER database contains epidemiological characteristics, primary tumor characteristics, stages, treatment options, and follow‐up information for multiple tumors.^27^ 33 Patients enrolled in the external validation of risk classification system were obtained from the First Affiliated Hospital of China Medical University between January 2019 and December 2021.

We screened out breast mucinous carcinoma (MBC) (ICD‐O‐3: 8480/3) diagnosed between 1988 and 2016 with SEER*Stat 8.3.9 software. The following information was further extracted from SEER: sex, race, marital status, age, tumor size, tumor grade, axillary lymph nodes examined, axillary positive lymph nodes, molecular subtype, estrogen receptor status (ER), progesterone receptor status (PR), human epidermal growth factor receptor 2 status (HER2), chemotherapy, radiotherapy, and follow‐up information. Based on the tumor characteristics and follow‐up information, we excluded the following patients: (1) male patients; (2) patients diagnosed with more than 1 malignant primary tumor; (3) patients who died within 3 months after surgery or lacked exact survival information; and (4) patients with unknown lymph node status. Subsequently, we included patients with complete information to perform the nomogram and axillary lymph node surgery analysis.

### 2.2 Statistical analysis

The chi‐square test was applied to compare the clinical categorical data. Comparisons of the tumor size and risk predictive score were analyzed by rank‐sum tests. Overall survival and breast cancer‐specific survival analyses were performed with the Kaplan–Meier method and the log‐rank test. Two‐sided *p* values of <0.05 were considered to be statistically significant.

A total of 655 patients with complete clinical information (age, marital status, race, grade, tumor size, positive lymph node, ER, PR, and HER2) were used to identify independent predictors of lymph node metastasis and to develop a nomogram model. Predictors of lymph node metastasis were identified with binomial logistic regression analysis with a backward stepwise procedure. Subsequently, independent predictors of tumor size, tumor grade, and age were used to develop a nomogram for predicting lymph node metastasis. Then, the predictive accuracy of the nomogram was validated using ROC analysis and quantified by AUC. Furthermore, a calibration plot was generated by bootstrapping with 1000 replications. Clinical usefulness and net benefit were estimated with decision curve analysis. Another 1018 patients with complete information on age, grade, tumor size, and axillary lymph node surgery approach were enrolled in the following risk classification analysis. We calculated the predictive score of each patient based on the nomogram with the “nomogramEx” package. Subsequently, a quartile was used to divide 1018 patients into four groups with three kinds of LNM risk.

Propensity score matching (PSM) analysis was conducted between the patients who underwent different axillary lymph node surgeries (no axillary surgery, sentinel lymph node biopsy, axillary lymph node detection). Variables of the predictive score, chemotherapy, and radiotherapy were selected for the propensity model to generate a matching ratio of 1:1. A *p* value <0.05 was used to estimate the balance between the variables before and after PSM.

PSM, nomogram construction, and validation were performed by R statistical software (version 3.6.3). Other analyses, including patient characteristic comparisons, survival analysis, and predictor identification, were performed with IBM SPSS statistical software (version 26.0).

## 3 RESULTS

### 3.1 Patient's selection and baseline characteristics

**FIGURE 1:**
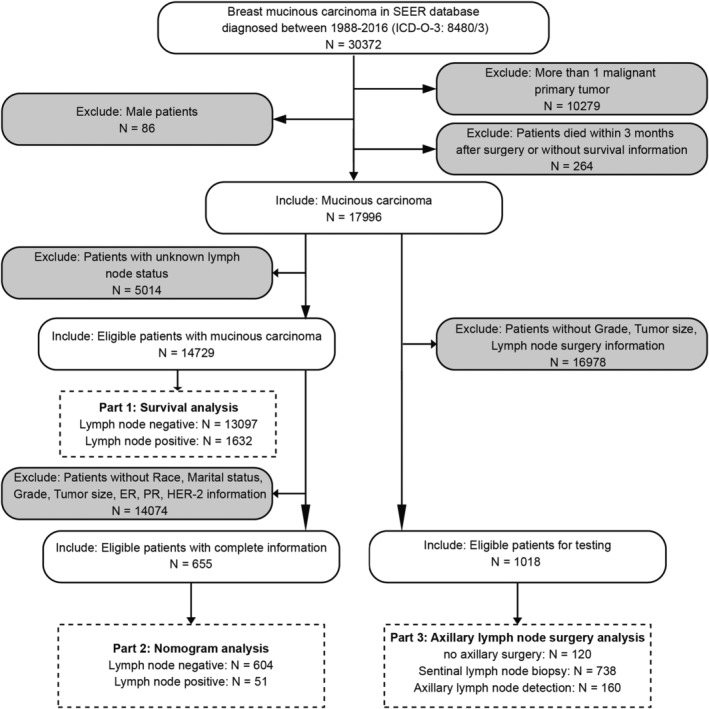
The flowchart of patients' selection. SEER, Surveillance Epidemiology and End Results

A total of 17,996 patients diagnosed with mucinous carcinoma were eligible for our study, including 14,729 patients who have intact lymph node information. Among 14,729 patients, 1632 patients (11.08%) had lymph node metastasis (LN+), and the other 13,097 patients (88.91%) were lymph node negative (LN‐). The epidemiologic characteristics, clinical features, and treatment strategies are summarized in Table 1. Patients with positive lymph nodes had a higher percentage with an age of less than 60 and a tumor size larger than 2 cm. The frequencies of ER and PR status were equal between the LN‐ and LN+ groups, while a higher percentage of HER2‐positive patients was observed in the LN+ group. Moreover, patients with lymph node metastasis were more likely to undergo partial mastectomy, axillary lymph node dissection (ALND), and chemotherapy treatment. In the subsequent analysis, we enrolled 655 patients out of 14,729 to perform nomogram model construction and validation. All 655 patients possessed complete information, including age, race, marital status, grade, tumor size, ER, PR, and HER2, which were eligible as independent predictors of lymph node metastasis. In addition, we selected 1018 out of 17,996 patients to further verify the nomogram model and to identify suitable surgical strategies for patients with different risks of lymph node metastasis. The patient selection flowchart for each analysis is displayed in Figure 1.

**TABLE 1:** The baseline characteristics of patients with mucinous breast carcinoma

Variables	LN negative (*N* = 13,097)	LN positive (*N* = 1632)	*p*
**Age**			< 0.001
< 40	528 (4.0%)	140 (8.6%)	
40–59	3743 (28.6%)	696 (42.6%)	
60–69	3261 (24.9%)	289 (17.7%)	
≥ 70	5565 (42.5%)	507 (31.1%)	
**Race**			< 0.001
White	10,339 (78.9%)	1209 (74.1%)	
Black	1184 (9.0%)	229 (14.0%)	
Others	1516 (11.6%)	189 (11.6%)	
Unknown	58 (0.4%)	5 (0.3%)	
**Marital status**			< 0.001
Married	6566 (50.1%)	788 (48.3%)	
Singled	1753 (13.4%)	306 (18.8%)	
Others	4275 (32.6%)	470 (28.8%)	
Unknown	503 (3.8%)	68 (4.2%)	
**AJCC‐TNM**			< 0.001
I	2657 (20.3%)	43 (2.6%)	
II	1088 (8.3%)	235 (14.4%)	
III	24 (0.2%)	152 (9.3%)	
IV	2 (0.0%)	22 (1.3%)	
Unknown	9326 (71.2%)	1180 (72.3%)	
**Tumor size**			< 0.001
≤ 2 cm	599 (4.6%)	27 (1.7%)	
2–5 cm	255 (1.9%)	35 (2.1%)	
> 5 cm	30 (0.2%)	20 (1.2%)	
Unknown	12,213 (93.3%)	1550 (95.0%)	
**Grade**			< 0.001
I	6375 (48.7%)	489 (30.0%)	
II	3624 (27.7%)	637 (39.0%)	
III	390 (3.0%)	193 (11.8%)	
IV	39 (0.3%)	12 (0.7%)	
Unknown	2669 (20.4%)	301 (18.4%)	
**ER**			< 0.001
Positive	11,404 (87.1%)	1411 (86.5%)	
Negative	299 (2.3%)	75 (4.6%)	
Borderline	14 (0.1%)	5 (0.3%)	
Unknown	1380 (10.5%)	141 (8.6%)	
**PR**			< 0.001
Positive	10,141 (77.4%)	1173 (71.9%)	
Negative	1309 (10.0%)	287 (17.6%)	
Borderline	70 (0.5%)	10 (0.6%)	
Unknown	1577 (12%)	162 (9.9%)	
**HER2**			< 0.001
Positive	199 (1.5%)	65 (4.0%)	
Negative	4087 (31.2%)	428 (26.2%)	
Borderline	62 (0.5%)	16 (1.0%)	
Unknown	8749 (66.8%)	1123 (68.8%)	
**Molecular subtype**			< 0.001
HR+/Her2‐	4062 (31.0%)	424 (26.0%)	
HR+/Her2+	177 (1.4%)	59 (3.6%)	
HR–/Her2+	22 (0.2%)	6 (0.4%)	
Triple negative	20 (0.2%)	4 (0.2%)	
Unknown	8816 (67.3%)	1139 (69.8%)	
**Breast surgery**			< 0.001
No surgery	8 (0.1%)	14 (0.9%)	
Partial mastectomy	833 (6.4%)	55 (3.4%)	
Total mastectomy	1081 (8.3%)	171 (10.5%)	
Unknown	11,175 (85.3%)	1392 (85.3%)	
**LN surgery**			< 0.001
NAS	7 (0.1%)	49 (3.0%)	
SLNB	7769 (59.3%)	367 (22.5%)	
ALND	5209 (39.8%)	1187 (72.7%)	
Unknown	112 (0.9%)	29 (1.8%)	
**Radiotherapy**			0.001
Yes	6663 (50.9%)	760 (46.6%)	
No	6434 (49.1%)	872 (53.4%)	
**Chemotherapy**			< 0.001
Yes	1321 (10.1%)	889 (54.5%)	
No	11,776 (89.9%)	743 (45.5%)	

Abbreviations: LN, lymph node; NAS, no axillary surgery; SLNB, sentinel lymph node biopsy; ALND, axillary lymph node dissection.

*p* < 0.05 was considered statistically significant.

### 3.2 Prognostic value of lymph node metastasis in MBC

**FIGURE 2:**
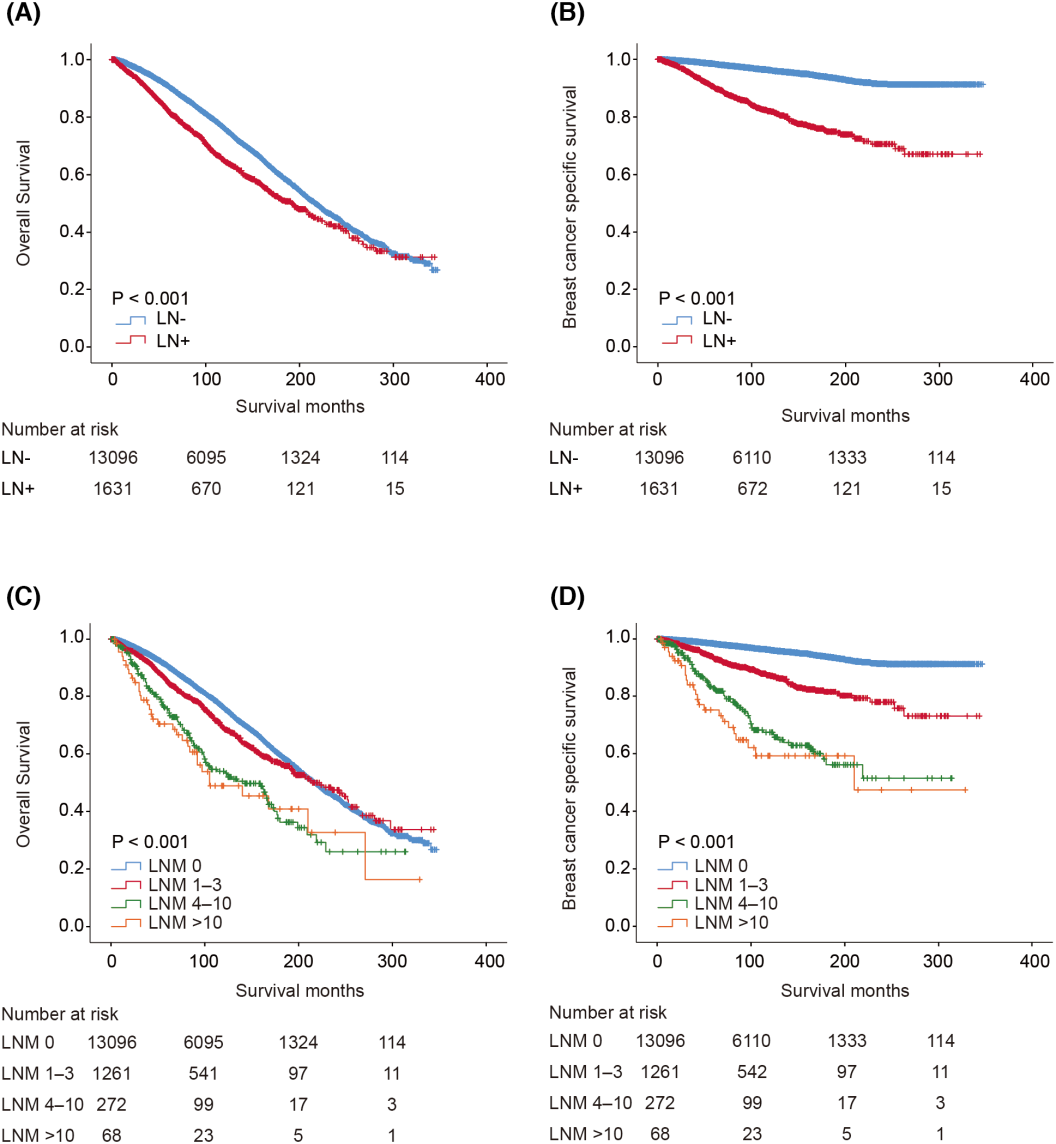
Survival analysis of mucinous breast carcinoma according to lymph node metastasis’. Kaplan–Meier curve of overall survival (A) and breast cancer‐specific survival (B) in patients with and without lymph node metastasis; Kaplan–Meier curve of overall survival (C) and breast cancer‐specific survival (D) in patients with different stage of lymph node metastasis. LN‐, no metastatic lymph node; LN+, exist metastatic lymph node; LNM, number of metastatic lymph nodes

The median follow‐up time of mucinous carcinoma was 101.72 months. The 5‐year and 10‐year overall survival (OS) rates in the LN‐negative and LN‐positive groups were 90.9% versus 82.4% and 76.1% versus 64.9%, respectively. The 5‐year and 10‐year breast cancer‐specific survival (BCSS) rates in the LN‐negative and LN‐positive groups were 98.4% versus 90.3% and 96.2% versus 82.0%, respectively. Therefore, patients with lymph node metastasis had worse survival than those without lymph node metastasis (*p* < 0.001) (Figure 2A–B). After adjusting for significant prognostic variables (age, grade, tumor size, marital status, race, ER, PR, HER2, radiotherapy, chemotherapy) in the univariate analysis, multivariate analysis indicated that the overall mortality risk and breast cancer‐specific death risk were higher in the LN‐positive group than in the LN‐negative group (OS: HR = 1.743, 95% CI: 1.576–1.928, *p* < 0.001; BCSS: HR = 3.783, 95% CI: 3.143–4.553, *p* < 0.001).

Additionally, we observed that OS was better for patients who had fewer than 3 metastatic lymph nodes than for those who had more than 4 metastatic lymph nodes (Figure 2C). The 10‐year OS rates were 76.1%, 69.0%, 54.0%, and 48.9% for the negative lymph node group (LN‐) and positive lymph node groups 1 (LNM 1–3), 2 (LNM 4–10), and 3 (LNM > 10), respectively. Consistent with OS, the BCSS was also better for patients with fewer metastatic lymph nodes, especially for patients who had no metastatic lymph nodes (Figure 2D). The 10‐year BCSS rates were 96.2%, 87.4%, 67.4%, and 59.2% for the negative lymph node group (LN‐) and positive lymph node groups 1 (LNM 1–3), 2 (LNM 4–10), and 3 (LNM > 10), respectively.

### 3.3 Identification of risk factors for lymph node metastasis

Considering that lymph node metastasis is crucial for mucinous carcinoma survival, we further identified effective risk factors related to lymph node metastasis. As shown in Table 2, univariate logistic analysis indicated that age, tumor size, grade, and HER2 were associated with lymph node metastasis. Further multivariate logistic analysis confirmed that age ≥ 70 (OR = 0.084, 95% CI: 0.012–0.578, *p* = 0.012), tumor size (OR = 1.037, 95% CI: 1.022–1.052, *p* < 0.001), grade II (OR = 2.526, 95% CI: 1.315–4.853, *p* = 0.005), and grade III/IV (OR = 4.245, 95% CI: 1.171–15.388, *p* = 0.028) were independent risk factors for lymph node metastasis. However, race, marital status, ER, and PR were not significantly correlated with lymph node metastasis in mucinous carcinoma.

**TABLE 2:** Univariate and multivariate analyses of predictive factors for lymph node metastasis

Variables	Univariable OR (95% CI)	*p*	Multivariable OR (95% CI)	*p*
**Age**				
≤ 29	Reference		Reference	
30–39	0.556 (0.078–3.965)	0.558	0.690 (0.088–5.390)	0.724
40–49	0.367 (0.060–2.252)	0.279	0.577 (0.087–3.835)	0.569
50–59	0.185 (0.030–1.139)	0.069	0.245 (0.037–1.636)	0.147
60–69	0.161 (0.027–0.954)	0.044	0.235 (0.037–1.489)	0.124
≥ 70	0.070 (0.011–0.441)	0.005	0.084 (0.012–0.578)	0.012
**Race**				
White	Reference			
Black	0.675 (0.233–1.955)	0.469		
Others	1.611 (0.806–3.221)	0.177		
**Marital status**				
Married	Reference			
Singled	0.965 (0.456–2.042)	0.925		
Others	0.555 (0.272–1.132)	0.105		
**Tumor Size**	1.036 (1.022–1.051)	< 0.001	1.037 (1.022–1.052)	< 0.001
**Grade**				
I	Reference		Reference	
II	2.520 (1.354–4.687)	0.004	2.526 (1.315–4.853)	0.005
III/ IV	8.652 (2.736–27.357)	< 0.001	4.245 (1.171–15.388)	0.028
**ER**				
Positive	Reference			
Negative	1.078 (0.136–8.522)	0.943		
**PR**				
Positive	Reference			
Negative	0.921 (0.319–2.660)	0.879		
Borderline	–	–		
**HER2**				
Positive	Reference			
Negative	0.224 (0.091–0.556)	0.001		
Borderline	1.179 (0.244–5.700)	0.838		

*p* < 0.05 was considered statistically significant.

### 3.4 Nomogram construction and validation for predicting lymph node metastasis

**FIGURE 3:**
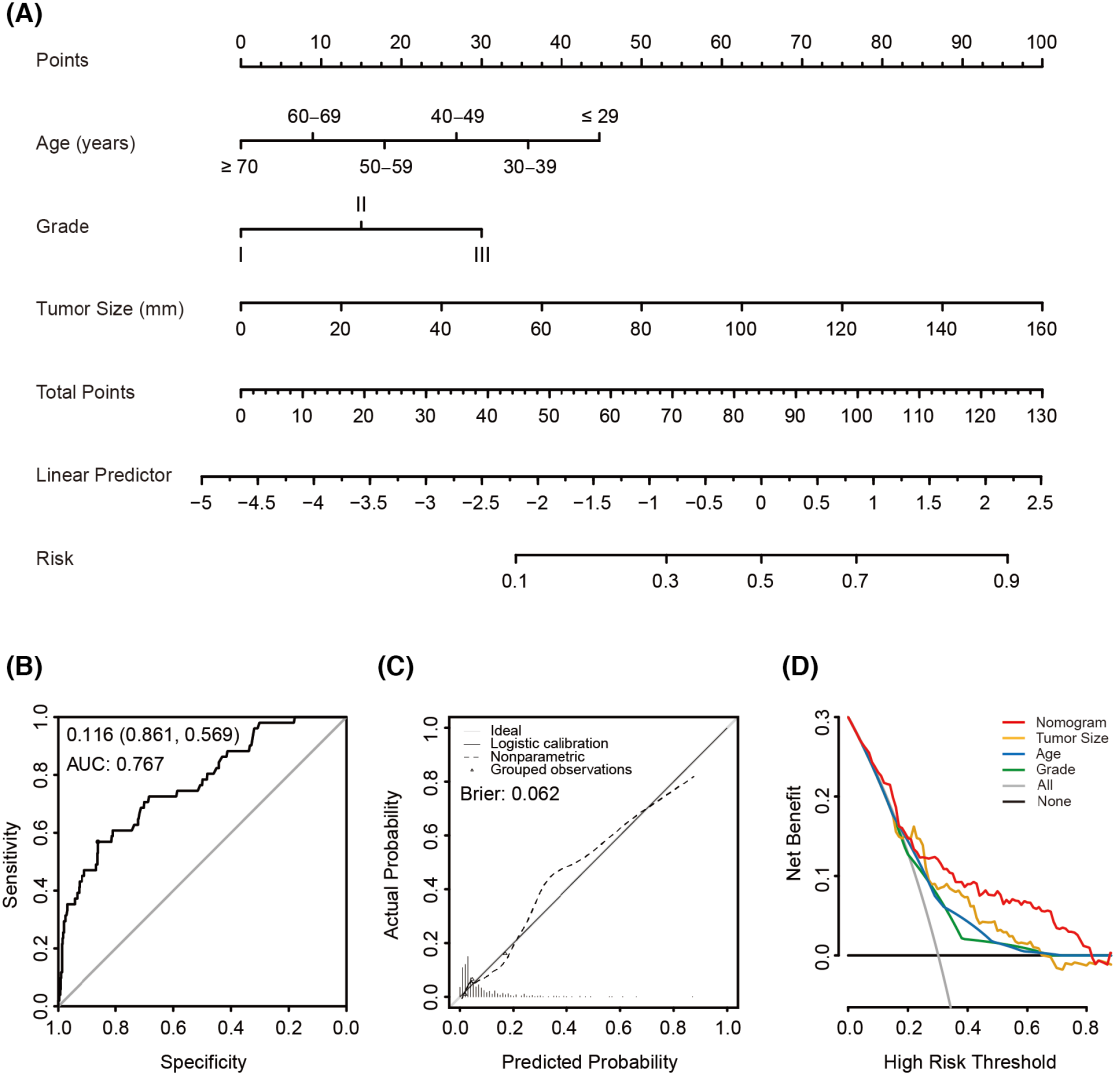
Construction and validation of nomogram for predicting the risk of lymph node metastasis of patients with mucinous breast carcinoma. (A) Nomogram for predicting lymph node metastasis of MBC. The value of each factor corresponds to a point at the top of the graph, the sum value of all the factors corresponds to a total point, and the bottom line corresponding vertically to the total point is the probability of lymph node metastasis. (B) ROC for discrimination of the nomogram, the AUC of the nomogram was 0.767 (95%CI: 0.569–0.861). (C) Calibration plot for the nomogram, x‐axis represents the nomogram predicted probabilities as measured by logistic regression analysis; y‐axis represents the actual probabilities, the dotted line represents the predictive power of the nomogram. (D) Decision curve for prediction of lymph node metastasis in MBC. Gray line, assume all patients will have lymph node metastasis; Black line, assume no patient will have lymph node metastasis. Red line, binary rule based on nomogram model; Yellow line, binary rule based on tumor size; Blue line, binary rule based on age; Green line, binary rule based on tumor grade

A nomogram was constructed from the independent risk factors for age, tumor size, and grade to predict the lymph node status (Figure 3A). The nomogram indicated good performance for evaluating lymph node metastasis with an AUC of 0.767 (95% CI: 0.697–0.838), and the sensitivity, specificity, and accuracy were 56.8%, 86.1%, and 83.8%, respectively (Figure 3B). The Hosmer–Lemeshow test also yielded a nonsignificant result (*p* = 0.263), which indicated that the nomogram is acceptable. The calibration plot also demonstrated good agreement between the bias‐corrected prediction and the ideal reference line with an additional 1000 bootstraps (mean absolute error = 0.062) (Figure 3C). In addition, compared to other prediction methods with a single factor alone (tumor size, grade, age), the nomogram provided the largest net benefit across the range of lymph node metastatic risk (Figure 3D).

### 3.5 Risk classification with nomogram and axillary surgery exploration

**FIGURE 4:**
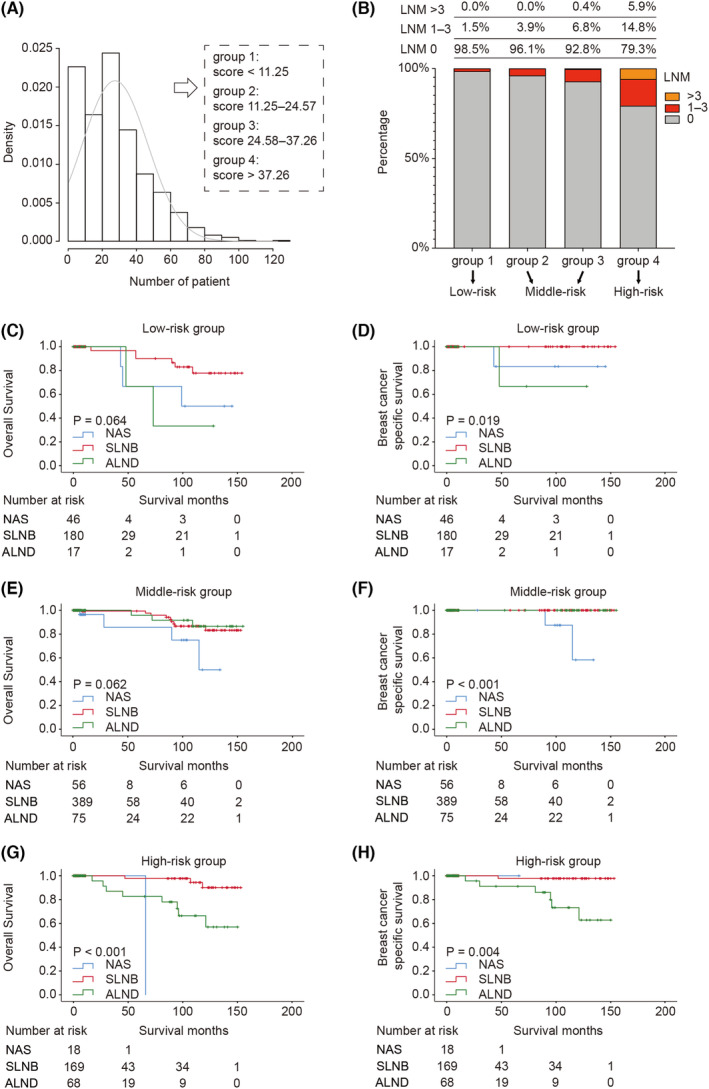
Risk classification and survival analysis of patients diagnosed with different lymph node metastasis risk. (A) Distribution curve of predictive score in 1018 patients. (B) Risk classification based on actually lymph node metastasis rate in each group. (C, D) Kaplan–Meier curve of overall survival and breast cancer‐specific survival in low‐risk patients treated with diverse axillary lymph node surgeries. (E, F) Kaplan–Meier curve of overall survival and breast cancer‐specific survival in middle‐risk patients treated with diverse axillary lymph node surgeries. (G, H) Kaplan–Meier curve of overall survival and breast cancer‐specific survival in high‐risk patients treated with diverse axillary lymph node surgeries. NAS, no axillary surgery; SLNB, sentinel lymph node biopsy; ALND, axillary lymph node dissection

To better classify patients with different risks of lymph node metastasis and to identify safe axillary surgery strategies with fewer complications, we screened out 1018 patients who have intact information of age, tumor size, grade, and axillary lymph node surgery. Subsequently, we grouped the 1018 eligible patients into four groups with the nomogram model (group 1: score < 11.25; group 2: score 11.25–24.57; group 3: 24.58–37.26; group 4: score > 37.26) (Figure 4A). Considering that few patients (1.5%) in group 1 had lymph node metastasis, we classified these patients as the low‐risk group. However, in group 4, 5.9% of patients had more than three metastatic lymph nodes, which indicated a poor prognosis. We considered these patients to be the high‐risk group. Compared to group 1 and group 4, patients in group 2 and group 3 had no more than 3 metastatic lymph nodes; therefore, these patients were classified into the middle‐risk group (Figure 4B).

According to the risk classification system, we performed external validation with 33 clinical node‐negative patients who were diagnosed with MBC and received axillary surgery in the First Affiliated Hospital of China Medical University. Through analysis, we observed that one patient who has three metastatic lymph nodes was identified as high‐risk, and two patients who have one metastatic lymph node were identified as middle‐risk and high‐risk separately (Table 3). Therefore, we proposed that the risk classification system was valid for clinical lymph node assessment of MBC.

**TABLE 3:** External validation of the risk classification system

Sample ID	Age	Tumor size	Grade	Risk score	Risk group	LNM
1	54	26	1	34.147169	Middle	0
2	64	25.9	1	25.136085	Middle	0
3	43	41.5	2	67.775913	high	0
4	39	22.2	2	64.661997	High	0
5	51	19.4	1	30.022169	Middle	0
6	92	40.2	1	25.125001	Middle	0
7	37	12.4	1	43.544337	High	0
8	67	16.8	1	19.448585	Middle	0
9	50	19.8	1	30.272169	Middle	0
10	52	68.1	1	60.459669	High	0
11	65	13.9	1	17.636085	Middle	0
12	50	26.7	1	34.584669	Middle	0
13	56	16.8	1	28.397169	Middle	0
14	29	28	1	62.242921	High	0
15	57	67.3	1	59.959669	High	0
16	53	26.7	1	34.584669	Middle	0
17	56	18.8	1	29.647169	Middle	0
18	48	36.4	1	49.595753	High	0
19	35	34	1	57.044337	High	3
20	42	18.6	1	38.470753	High	0
21	47	21.2	1	40.095753	High	1
22	44	35.6	1	49.095753	High	0
23	70	38.2	1	23.875001	Middle	0
24	78	26.8	1	16.750001	Middle	0
25	50	20.7	1	30.834669	Middle	0
26	71	99	1	61.875001	High	0
27	69	17.7	1	20.011085	Middle	0
28	61	22.8	1	23.198585	Middle	0
29	61	40.9	1	34.511085	Middle	1
30	61	22.8	1	23.198585	Middle	0
31	57	56.7	1	53.334669	High	0
32	40	25	1	42.470753	High	0
33	68	29.9	1	27.636085	Middle	0

Abbreviations: LNM, the number of metastatic lymph node in axillary surgery.

Based on the risk classification, we further explored the survival benefits of different axillary lymph node surgeries in the low‐, middle‐, and high‐risk groups separately. The clinical features and axillary treatments in each cohort are summarized in Table 4. Through survival analysis, we observed that in the low‐risk group, the OS and BCSS of SLNB were better than those of no axillary surgery (NAS) and ALND (OS: *p* = 0.064; BCSS: *p* = 0.019) (Figure 4C–D). In the middle‐risk group, the OS and BCSS of SLNB were similar to those of ALND, and both treatments were superior to NAS (OS: *P* = 0.062; BCSS: *P* < 0.001) (Figure 4E–F). In the high‐risk group, SLNB was significantly superior to ALND (OS: *p* < 0.001; BCSS: *p* = 0.004) (Figure 4G–H).

**TABLE 4:** The baseline characteristics of patients in low‐risk, middle‐risk, and high‐risk cohorts

Variables	Low‐risk group (*N* = 243)				Middle‐risk group (*N* = 520)				High‐risk group (*N* = 255)			
	NAS (*N* = 46)	SLNB (*N* = 180)	ALND (*N* = 17)	*p*	NAS (*N* = 56)	SLNB (*N* = 389)	ALND (*N* = 75)	*p*	NAS (*N* = 18)	SLNB (*N* = 169)	ALND (*N* = 68)	*p*
**Predictive score** (median [inter‐quatile range])	6.56 (3.28)	6.56 (4.37)	7.50 (4.06)	0.248	20.31 (11.09)	24.77 (10.00)	24.99 (10.62)	0.005*	50.71 (39.20)	49.76 (15.31)	54.99 (24.78)	0.005*
**Tumor Size**				–				0.218				< 0.001*
≤ 2 cm	46 (100%)	108 (100%)	17 (100%)		33 (58.9%)	256 (65.8%)	39 (52.0%)		3 (16.7%)	75 (44.4%)	14 (20.6%)	
2–5 cm	–	–	–		22 (39.3%)	129 (33.2%)	35 (46.7%)		4 (22.2%)	79 (46.7%)	30 (44.1%)	
> 5 cm	–	–	–		1 (1.8%)	4 (1.0%)	1 (1.3%)		11 (61.1%)	15 (8.9%)	24 (35.3%)	
**Age**				–				0.002*				0.003*
≤ 29	–	–	–		–	–	–		0 (0.0%)	6 (3.6%)	2 (2.9%)	
30–39	–	–	–		0 (0.0%)	2 (0.5%)	1 (1.3%)		4 (22.2%)	28 (16.6%)	6 (8.8%)	
40–49	–	–	–		2 (3.6%)	38 (9.8%)	1 (1.3%)		0 (0.0%)	50 (29.6%)	21 (30.9%)	
50–59	–	–	–		2 (3.6%)	83 (21.3%)	13 (17.3%)		3 (16.7%)	55 (32.5%)	22 (32.4%)	
≥ 70	46 (100%)	108 (100%)	17 (100%)		52 (92.9%)	266 (68.4%)	60 (80.0%)		11 (61.1%)	30 (17.8%)	17 (25.0%)	
**Grade**				–				0.048*				0.044*
I	46 (100%)	108 (100%)	17 (100%)		24 (42.9%)	203 (52.2%)	48 (64.0%)		10 (55.6%)	40 (23.7%)	20 (29.4%)	
II	–	–	–		32 (57.1%)	186 (47.8%)	27 (36.0%)		8 (44.4%)	104 (61.5%)	40 (58.8%)	
III	–	–	–		–	–	–		0 (0.0%)	25 (14.8%)	8 (11.8%)	
**Chemotherapy**				0.037*				0.226				0.013*
Yes	1 (2.2%)	3 (1.7%)	2 (11.8%)		3 (5.4%)	26 (6.7%)	9 (12.0%)		3 (16.7%)	58 (34.3%)	34 (50.0%)	
No	45 (97.8%)	177 (98.3%)	15 (88.2%)		53 (94.6%)	363 (93.3%)	66 (88.0%)		15 (83.3%)	111 (65.7%)	34 (50.0%)	
**Radiotherapy**				< 0.001*				< 0.001*				0.001*
Yes	6 (13.0%)	104 (57.8%)	7 (41.2%)		12 (21.4%)	248 (63.8%)	35 (46.7%)		2 (11.1%)	97 (57.4%)	38 (55.9%)	
No	40 (87.0%)	76 (42.2%)	10 (58.8%)		44 (78.6%)	141 (36.2%)	40 (53.3%)		16 (88.9%)	72 (42.6%)	30 (44.1%)	

Abbreviations: NAS, no axillary surgery; SLNB, sentinel lymph node biopsy; ALND, axillary lymph node dissection.

^*^ *p*  < 0.05 was considered statistically significant.

### 3.6 Stratified analysis of OS within the matched cohort to identify the suitable axillary surgery for mucinous carcinoma with different risks

Patients who received radiotherapy and chemotherapy treatment may have been subject to some selection bias. Therefore, to adjust for potential baseline bias and confounders, we performed propensity score matching (PSM) in each group separately. Detailed information on the original cohort, unmatched cohort, and propensity‐matched cohort is shown in Table 5.

**TABLE 5:** The baseline characteristics of patients before and after propensity score matching

Variables	Low‐risk group						Middle‐risk group						High‐risk group					
	Original (*N* = 226)			After PSM (*N* = 90)			Original (*N* = 464)			After PSM (*N* = 142)			Original (*N* = 237)			After PSM (*N* = 100)		
	NAS (*N* = 46)	SLNB (*N* = 180)	*p*	NAS (*N* = 45)	SLNB (*N* = 45)	*p*	SLNB (*N* = 389)	ALND (*N* = 75)	*p*	SLNB (*N* = 71)	ALND (*N* = 71)	*p*	SLNB (*N* = 169)	ALND (*N* = 68)	*p*	SLNB (*N* = 50)	ALND (*N* = 50)	*p*
**Predictive score** (median [inter‐quatile range])	6.56 (3.28)	6.56 (4.38)	0.93	6.88 (3.13)	7.5 (3.75)	0.027*	24.77 (10.00)	24.99 (10.62)	0.124	24.74 (9.38)	24.99 (9.38)	0.321	49.76 (15.31)	54.99 (24.78)	0.001*	50.60 (17.59)	50.90 (15.48)	0.879
**Tumor Size**			–			–						0.167			< 0.001*			0.015*
≤ 2 cm	46 (100%)	180 (100%)		45 (100%)	45 (100%)		256 (65.8%)	39 (52.0%)		47 (66.2%)	36 (50.7%)		75 (44.4%)	14 (20.6%)		23 (46.0%)	14 (28.0%)	
2–5 cm	–	–		–	–		129 (33.2%)	35 (46.7%)		23 (32.4%)	34 (47.9%)		79 (46.7%)	30 (44.1%)		25 (50.0%)	25 (50.0%)	
> 5 cm	–	–		–	–		4 (1.0%)	1 (1.3%)		1 (1.4%)	1 (1.4%)		15 (8.9%)	24 (35.3%)		2 (4.0%)	11 (22.0%)	
**Age**			–			–			0.053			0.71			0.494			0.39
≤ 29	–	–		–	–		–	–		–	–		6 (3.6%)	2 (2.9%)		3 (6.0%)	1 (2.0%)	
30–39	–	–		–	–		2 (0.5%)	1 (1.3%)		0 (0.0%)	1 (1.4%)		28 (16.6%)	6 (8.8%)		7 (14.0%)	4 (8.0%)	
40–49	–	–		–	–		38 (9.8%)	1 (1.3%)		2 (2.8%)	1 (1.4%)		50 (29.6%)	21 (30.9%)		12 (24.0%)	15 (30.0%)	
50–59	–	–		–	–		83 (21.3%)	13 (17.3%)		14 (19.7%)	13 (18.3%)		55 (32.5%)	22 (32.4%)		19 (38.0%)	15 (30.0%)	
≥ 70	46 (100%)	180 (100%)		45 (100%)	45 (100%)		266 (68.4%)	60 (80.0%)		55 (77.5%)	56 (78.9%)		30 (17.8%)	17 (25%)		9 (18.0%)	15 (30.0%)	
**Grade**			–			–			0.076			0.496			0.604			0.044*
I	46 (100%)	180 (100%)		45 (100%)	45 (100%)		203 (52.2%)	48 (64.0%)		39 (54.9%)	44 (62.0%)		40 (23.7%)	20 (29.4%)		9 (18.0%)	17 (34.0%)	
II	–	–		–	–		186 (47.8%)	27 (36.0%)		32 (45.1%)	27 (38.0%)		104 (61.5%)	40 (58.8%)		31 (62.0%)	30 (60.0%)	
III	–	–		–	–		–	–		–	–		25 (14.8%)	8 (11.8%)		10 (20.0%)	3 (6.0%)	
**Chemotherapy**			1.00			–			0.147			0.166			0.025*			0.529
Yes	1 (2.2%)	3 (1.7%)		–	–		26 (6.7%)	9 (12.0%)		2 (2.8%)	7 (9.9%)		58 (34.3%)	34 (50.0%)		16 (32.0%)	19 (38.0%)	
No	45 (97.8%)	177 (98.3%)		45 (100%)	45 (100%)		363 (93.3%)	66 (88.0%)		69 (97.2%)	64 (90.1%)		111 (65.7)	34 (50.0%)		34 (68.0%)	31 (62.0%)	
**Radiotherapy**			< 0.001*			1.00			0.007*			0.867			0.831			0.689
Yes	6 (13.0%)	104 (57.8%)		40 (88.9%)	40 (88.9%)		248 (63.8%)	35 (46.7%)		33 (46.5%)	35 (49.3%)		97 (57.4%)	38 (55.9%)		27 (54.0%)	25 (50.0%)	
No	40 (87.0%)	76 (42.2%)		5 (11.1%)	5 (11.1%)		141 (36.2%)	40 (53.3%)		38 (53.5%)	36 (50.7%)		72 (42.6%)	30 (44.1%)		23 (46.0%)	25(50.0%)	

Abbreviations: NAS, no axillary surgery; SLNB, sentinel lymph node biopsy; ALND, axillary lymph node dissection.

^*^ *p*  < 0.05 was considered statistically significant.

**FIGURE 5:**
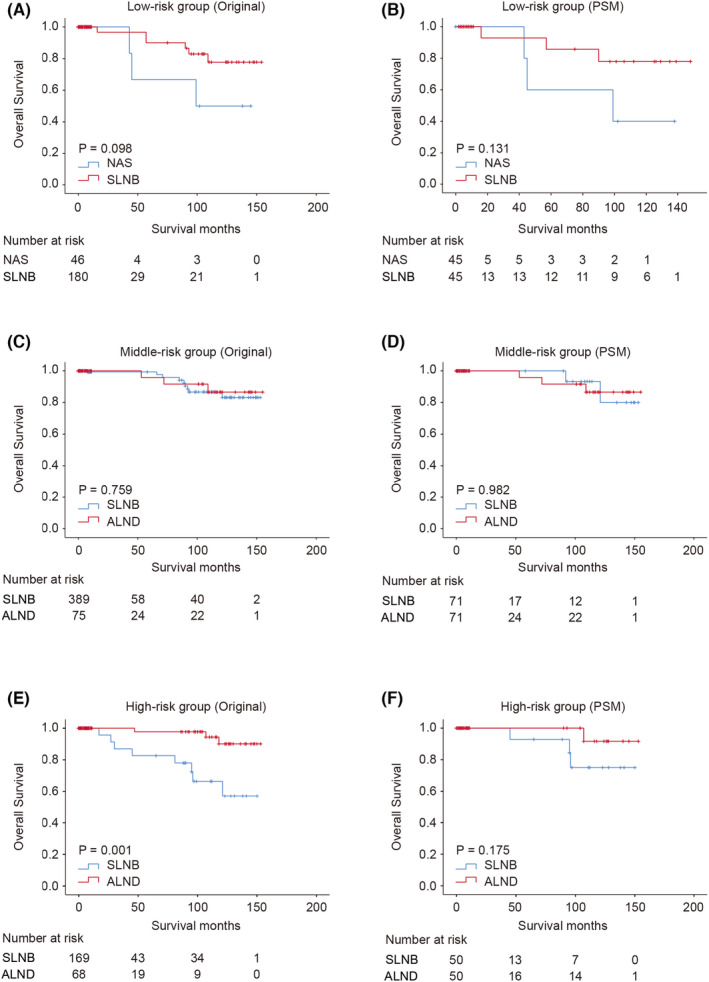
Survival analysis of mucinous breast carcinoma according to diverse axillary survival treatment. Kaplan–Meier curve of overall survival in low‐risk cohort patients treated with no axillary surgery and SLNB before (A) and after propensity score matching (B). Kaplan–Meier curve of overall survival in middle‐risk cohort patients treated with SLNB and ALND before (C) and after propensity score matching (D). Kaplan–Meier curve of overall survival in high‐risk cohort patients treated with sentinel lymph node biopsy (SLNB) and axillary before (E) and after propensity score matching (F). NAS, no axillary surgery; SLNB, sentinel lymph node biopsy; ALND, axillary lymph node dissection

After matching, the 5‐year and 10‐year OS rates of NAS and SLNB in the low‐risk group were 60% vs. 85.7%, 40% versus 77.9%, respectively, the 5‐year and 10‐year BCSS rates of NAS and SLNB in the low‐risk group were 80% vs. 100%, 80% vs. 100%. There were no statistically significant survival benefits of OS and BCSS in low‐risk group (OS: *p* = 0.131; BCSS: *p* = 0.107) (Figure 5A–B, Supplementary Figure S1A–B). Similar to the low‐risk group, the 5‐year and 10‐year OS rates of SLNB and ALND in the middle‐risk were 93.3% versus 95.8% and 80.0% versus 86.6%, respectively. The 5‐year and 10‐year OS rates of SLNB and ALND in the middle‐risk were all 100% versus 100%. No statistically significant survival benefits were observed between SLNB and ALND in the middle‐risk group (OS: *p* = 0.759, BCSS: *p* = 1) (Figure 5C–D). Meanwhile, survival analysis showed comparable OS and BCSS between patients underwent SLNB and ALND in high‐risk cohort (OS: *p* = 0.175; BCSS: *p* = 0.093). The 5‐year and 10‐year OS rates of SLNB and ALND in the high‐risk group were 100% versus 100% and 80.8% versus 100%, and the BCSS rates were 92.9% versus 100% and 75.0% versus 91.7% (Figure 5E–F, Supplementary Figure S1C–D).

## 4 DISCUSSION

Mucinous breast carcinoma (MBC) is a unique disease with a low incidence and favorable clinical characteristics.^5^, ^6^, ^7^, ^8^ Given its rarity, treatments for MBC are adopted from those for invasive breast cancer (IDC), and the survival benefits for patients remain ambiguous. Previous studies have demonstrated that lymph node status is a crucial factor for MBC prognosis.^6^, ^10^, ^11^, ^12^, ^13^ Therefore, estimating the risk of lymph node metastasis preoperatively and identifying suitable procedures for axillary surgery are necessary. In this study, we constructed and validated a nomogram to predict the lymph node metastasis risk of MBC with independent predictive factors of tumor size, age, and grade. Based on the nomogram model, we established a risk classification system and categorized patients into three groups with different lymph node metastasis risks. Subsequently, we investigated the optimal axillary surgery strategy in the low‐risk, middle‐risk, and high‐risk groups separately.

The probability of axillary lymph node metastasis in MBC is low; nonetheless, metastatic nodes are closely associated with a worse prognosis. Currently, lymph node status is mainly assessed by imaging examination, including ultrasound, mammography, and magnetic resonance imaging (MRI).^28^, ^29^, ^30^ Although ultrasound and MRI can show lymph node metastasis, only a portion of patients will undergo MRI detection, and the accuracy of ultrasound detection mainly relies on the sonographer's capability. Thus, we constructed a nomogram for predicting the risk of LNM with preoperative clinical characteristics, which can be conveniently obtained in clinical practice. Based on the nomogram model, surgeons can evaluate the risk of lymph node metastasis and then optimize the surgical strategy. In our study, tumor size, age, and grade were identified as independent factors for LNM and were included in the nomogram. Aside from the above three parameters, previous studies also reported that HER2‐positive PMBC shows more frequent lymph node metastasis and poor outcomes.^22^, ^23^, ^31^ In our study, HER2 status was related to lymph node metastasis in univariate analysis; however, no significant correlation was observed in the following multivariate analysis. We speculated that the discrepancy might be ascribed to the limited number of samples diagnosed with HER2 positivity in our study, and all HER2‐positive MBCs were mixed with infiltrating ductal carcinoma in previous reports.^32^

Based on the nomogram, we further stratified patients with predictive scores and successfully separated patients into three cohorts with different risks of LNM. Based on our classification, patients and surgeons can be made directly aware of the metastasis risk. Subsequently, we investigated the survival benefits of different axillary surgeries and optimized surgical treatment with high quality of life. In the axillary management of IDC, the strategy of axillary surgery is mainly based on tumor size.^33^ In our study, we observed that the prediction with the nomogram was more precise than that with a single risk factor. Thus, it might be possible to predict lymph node metastasis and choose an appropriate axillary surgical approach using the nomogram instead of tumor size.

Given that LNM indicates adverse outcomes, patients with LNM always receive more aggressive surgical treatment and additional adjuvant therapies, such as radiotherapy and chemotherapy. To date, existing studies have proposed that postoperative radiotherapy and chemotherapy are partly associated with a better prognosis of MBC.^16^, ^17^, ^18^ Therefore, to reduce the selection bias caused by adjuvant therapies, we performed propensity score matching (PSM) in each group. After PSM, no significant survival advantages of sentinel lymph node biopsy (SLNB) were observed compared to the NAS in the low‐risk patients. In recent decades, de‐escalation of axillary surgery in early breast cancer has been widely investigated, and omission of SLNB in elderly patients is currently discussed hot topic. Due to no significant benefit of axillary clearance in elderly patients in BCSG trial 10–93^34^ and Martelli's study,^35^ Society of Surgical Oncology (SSO) Choosing Wisely Guideline recommend against routine SLNB in clinically node‐negative (cN0), hormone receptor (HR) positive, HER2 negative patients with age ≥ 70.^36^ Furthermore, James Sun's study suggested that SLNB can be safely omitted in elderly patients with T1, HR‐positive breast cancer.^37^ Several studies^38^, ^39^ proposed that omission of axillary staging in elderly patients did not affect overall survival. In contrast to patients with N0, Vivienne's study proposed that it is rational to perform SLNB to elderly patients with one positive SLN, cause such patients were less likely to receive chemotherapy.^40^ In our study, all the patients allocated to the low‐risk group are age ≥ 70, with grade I, small tumor size (≤2 cm), and HR‐positive. Therefore, combined with negative clinical examinations, we proposed that it is relatively safe for such elderly patients who are assessed with clinical node‐negative and low‐risk scores to omit SLNB. While for patients with suspicious metastatic lymph node, it is necessary to perform SLNB. Similar to the low‐risk group, the use of SLNB did not result in inferior survival than axillary lymph node dissection (ALND) in the middle‐risk and high‐risk groups. Since the NSABP‐B32 trial, the treatment of ALND has been replaced by SLNB for patients with clinically node negative. Subsequently, in the post‐Z0011 era, ALND was not recommended for early‐stage breast cancer patients with 1–2 positive sentinel lymph nodes (SLNs).^41^, ^42^ Meanwhile, AMAROS trial^43^ and Edinburgh trial^44^ indicated that axillary radiotherapy could offer comparable regional control and survival benefits to ALND with fewer complications for patients with T1–2. In our study, almost all the patients in the middle‐risk group have relatively a small tumor (T1–2) and limited metastatic lymph nodes (≤3). Therefore, we proposed that SLNB is adequate for most of the patients in the middle‐risk group, and SLNB plus radiotherapy is an effective choice for patients with a tumor size ≤5 cm and positive SLNs ≤2 in the middle‐ and high‐risk groups. Regarding patients with more than three positive SLNs in the middle and high‐risk groups, ALND could provide more information on axillary staging which affects systematic treatment. Therefore, patients in the middle and high‐risk group, who are candidate for additional adjuvant treatment should be considered for ALND.

There are several limitations of our study. Given the rarity of MBC, a single center could not obtain enough cases; therefore, the predictive model and the risk classification system in our study were based on data obtained from the SEER database. In this study, HER2 was not necessarily associated with LNM and therefore was not included in the nomogram. Regarding the correlation between HER2 and LNM in previous reports, studies based on more samples will be needed in the future to verify the necessity of HER2 inclusion in the nomogram. In the SEER database, information on axillary recurrence, adjuvant endocrine therapy, and local treatment of primary tumors is not available; therefore, we could not assess the recurrence risk of MBC and could not include these therapeutic factors in the PSM. Additionally, although the accuracy of the nomogram model and the risk classification system were satisfactory, further verification of the nomogram model and risk classification system with more clinical data is necessary.

## 5 CONCLUSION

**FIGURE 6:**
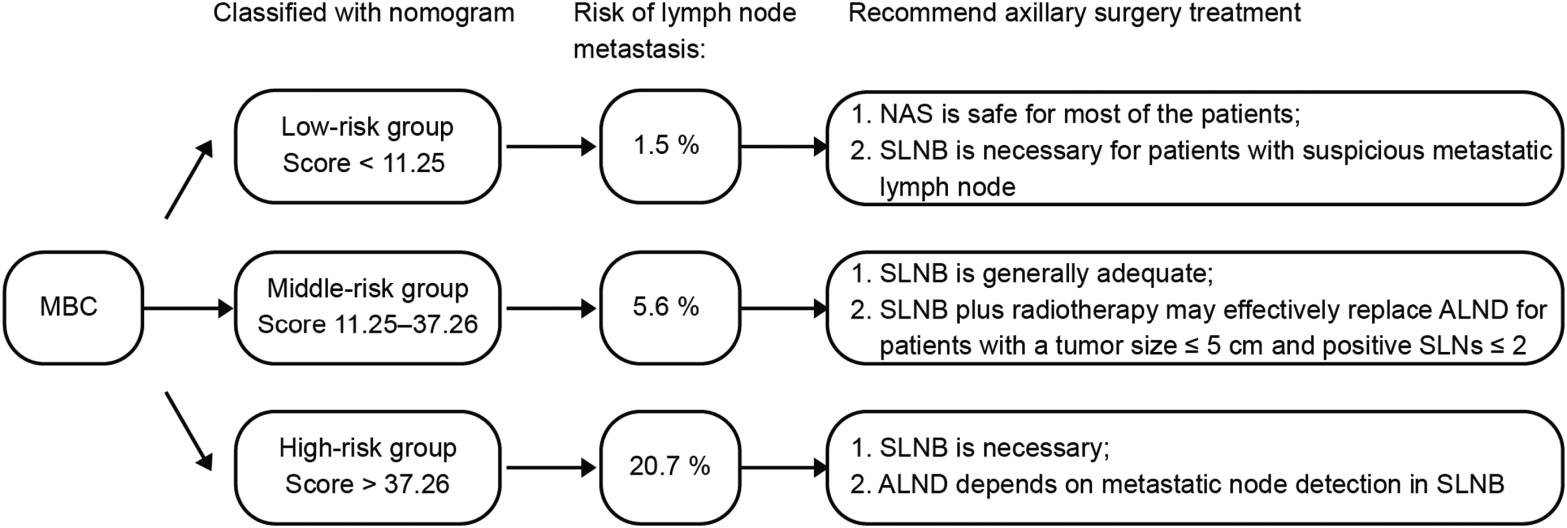
Summary of mucinous breast carcinoma's classification with diverse risk of lymph node metastasis based on nomogram and recommended axillary surgery treatment in each group. MBC, mucinous breast carcinoma; NAS, no axillary surgery; SLNB, sentinel lymph node biopsy; ALND, axillary lymph node dissection; SLNs, sentinel lymph nodes

According to the patient characteristics and survival analysis of the surgical treatments in different groups, we propose the following (Figure 6):

1. No axillary surgery is safe for most of the patients classified into the low‐risk group (age ≥ 70, tumor size ≤2 cm, and grade I). SLNB is necessary for patients who have suspicious metastatic lymph nodes in clinical detection.
2. SLNB is necessary for patients who are classified into the middle‐risk and high‐risk groups.

- Most of the patients in the middle‐risk cohort had fewer than three metastatic lymph nodes, SLNB is generally adequate for these patients.
- Given that most tumors in the middle‐risk group are T1‐T2 (≤5 cm), grade I/II and HR+, SLNB plus radiotherapy may effectively replace ALND for patients with a tumor size ≤5 cm, SLNs ≤2.
- Both SLNB and ALND are suitable for high‐risk patients. Detailed decisions about further surgery depend on metastatic node detection in SLNB.

## CONFLICT OF INTEREST

The authors declare that they have no competing interests.

## Ethics approval and consent to participate

We acknowledge the efforts of the Surveillance, Epidemiology, and End Results (SEER) Program registries for creating the SEER database. All procedures performed in studies involving human participants were in accordance with the ethical standards of the institutional and/or national research committee and with the 1964 Helsinki Declaration and its later amendments or comparable ethical standards. This study was approved by the Medical Scientific Research Ethics Committee of the First Affiliated Hospital of China Medical University.

## AUTHORS' CONTRIBUTION

SLW and JDG performed data acquisition, SLW contributed to data interpretation, statistical analysis, and drafting the manuscript. SLW, XMY, XYM and FJ contributed to the study design and data interpretation. All the authors contributed to the article and approved the manuscript.

## Funding information

This work was supported by grants from National Natural Science Foundation of China under Grant 82073282 and 81972791.

## Supporting information


Figure S1

## Data Availability

Data used for nomogram construction and survival analyses were extracted from the Surveillance Epidemiology and End Results (SEER) database released in November 2018. Qualified researchers may access to information on cancer statistics through the website of SEER database (https://seer.cancer.gov/). Data used for external validation were extracted from the First Affiliated Hospital of China Medical University and are available in Table 3, and detailed information is available from the corresponding author on reasonable request.
