# Supplementary figures and images for: A novel nomogram and risk classification system for predicting lymph node metastasis of breast mucinous carcinoma: A SEER‐based study
Source: Cancer Med. 2022 May 22;11(24):4767–83. doi: 10.1002/cam4.4804 (PMC9761057; doi:10.1002/cam4.4804)

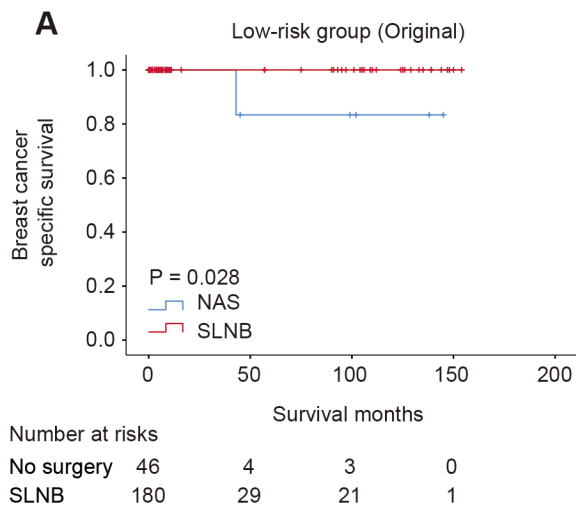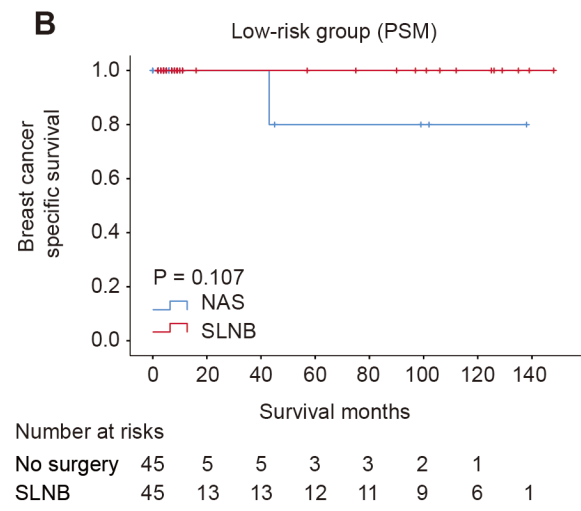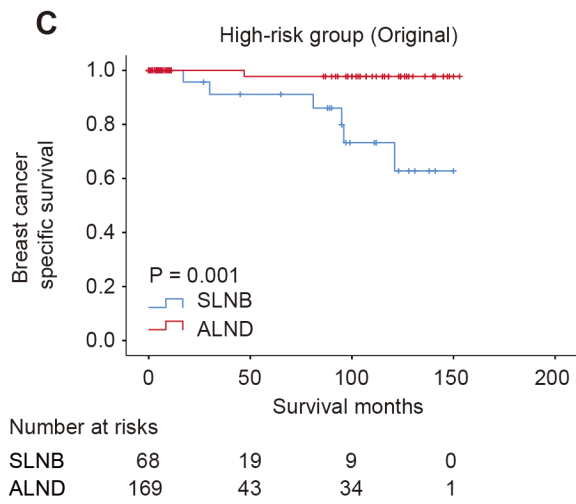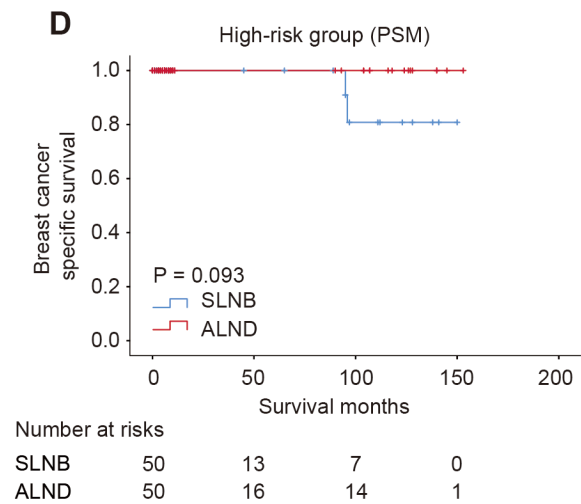

Supplement: Supplementary file 1 — Figure S1 [file CAM4-11-4767-s001.pdf]
